# Angina Pectoris, or Diaphragmatic Neuralgia

**Published:** 1831

**Authors:** 


### XLVI.

### ANGINA PECTORIS, OR DIAPHRAGMATIC NEURALGIA.

A MAN, 37 years of age, of sanguineous temperament, had long been subject to attacks of dyspnœa, and palpitation, for which he was now received into the Hôtel Dieu of Paris. These symptoms had commenced when he was about sixteen years of age, and they were attended by acute pain in the situation of the diaphragm and of the heart. They were easily excited or aggravated by any muscular exertion. Notwithstanding this state of health, he was drawn as a conscript, and served at the battle of Wagram, where he was wounded, and then discharged. At the age of 23, the year after his discharge, he had a severe attack of hæmoptysis, which seemed to relieve the other symptoms. Next year he had a similar attack, and with similar results. Having taken to the occupation of silvering mirrors, he suffered from mercurial emanations, and experienced numbness of the upper extremities, and many symptoms of indigestion. The spasmodic constrictions about the heart and diaphragm, however, were the most distressing part of the complaint, and for these chiefly, he entered the hospital. Two days elapsed before he was examined, and this interval of quietude had greatly calmed the action of the heart, which was nearly natural, as was the pulse. The chest was quite sonorous in every part—and the respiration was every where audible. Nothing wrong about the heart could be detected by the stethoscope. The tongue was clean, and pressure on the abdomen was easily borne, except at the epigastrium, where sudden pressure upwards brought on all the symptoms of angina pectoris. It was now observed that although the chest was well formed in respect to breadth, yet it was very much the contrary in respect to depth. Although sudden pressure at the epigastrium excited the most distressing symptoms, it was ascertained that a gradual and uniform pressure on the whole of the abdomen, by means of a roller, gave ease and comfort to the patient. The man was put upon light diet, had eight ounces of blood taken from his arm, and was enjoined quietude. In a short time he was able to ascend a pair of stairs without much inconvenience, and in a few weeks he left the hospital — certainly not cured, but much better than when he entered the institution. The reporter, by a long train of reasoning, which we shall not follow, and cannot entirely admit, comes to the conclusion that this was a case of diaphragmatic neuralgia. We doubt whether the great distress produced by pressure on the epigastrium be any proof of the said neuralgia.

---

